# Minigene Splicing Assays and Long-Read Sequencing to Unravel Pathogenic Deep-Intronic Variants in *PAX6* in Congenital Aniridia

**DOI:** 10.3390/ijms24021562

**Published:** 2023-01-13

**Authors:** Alejandra Tamayo, Gonzalo Núñez-Moreno, Carolina Ruiz, Julie Plaisancie, Alejandra Damian, Jennifer Moya, Nicolas Chassaing, Patrick Calvas, Carmen Ayuso, Pablo Minguez, Marta Corton

**Affiliations:** 1 Department of Genetics & Genomics, Instituto de Investigación Sanitaria-Fundación Jiménez Díaz University Hospital, Universidad Autónoma de Madrid (IIS-FJD, UAM), 28040 Madrid, Spain; 2 Center for Biomedical Network Research on Rare Diseases (CIBERER), Instituto de Salud Carlos III, 28029 Madrid, Spain; 3 Department of Surgery, Medical and Social Sciences, Faculty of Medicine and Health Sciences, Science and Technology Campus, University of Alcalá, 28871 Alcalá de Henares, Spain; 4 Bioinformatics Unit, Instituto de Investigación Sanitaria-Fundación Jiménez Díaz University Hospital, Universidad Autónoma de Madrid (IIS-FJD, UAM), 28240 Madrid, Spain; 5 Centre de Référence des Affections Rares en Génétique Ophtalmologique (CARGO), Hôpital Purpan, CHU Toulouse, 31000 Toulouse, France; 6 INSERM U1214, Université Toulouse III, 31000 Toulouse, France

**Keywords:** *PAX6*, congenital aniridia, non-canonical splicing sites, deep-intronic variants, minigene splicing assays, long-read sequencing, MinION nanopore sequencing

## Abstract

*PAX6* haploinsufficiency causes aniridia, a congenital eye disorder that involves the iris, and foveal hypoplasia. Comprehensive screening of the *PAX6 locus*, including the non-coding regions, by next-generation sequencing revealed four deep-intronic variants with potential effects on pre-RNA splicing. Nevertheless, without a functional analysis, their pathogenicity could not be established. We aimed to decipher their impact on the canonical *PAX6* splicing using in vitro minigene splicing assays and nanopore-based long-read sequencing. Two multi-exonic *PAX6* constructs were generated, and minigene assays were carried out. An aberrant splicing pattern was observed for two variants in intron 6, c.357+136G>A and c.357+334G>A. In both cases, several exonization events, such as pseudoexon inclusions and partial intronic retention, were observed due to the creation or activation of new/cryptic non-canonical splicing sites, including a shared intronic donor site. In contrast, two variants identified in intron 11, c.1032+170A>T and c.1033-275A>C, seemed not to affect splicing processes. We confirmed the high complexity of alternative splicing of *PAX6* exon 6, which also involves unreported cryptic intronic sites. Our study highlights the importance of integrating functional studies into diagnostic algorithms to decipher the potential implication of non-coding variants, usually classified as variants of unknown significance, thus allowing variant reclassification to achieve a conclusive genetic diagnosis.

## 1. Introduction

Congenital aniridia (MIM #106210) is a rare panocular disease with an estimated worldwide incidence of about 1 per 50,000 to 100,000 births [1]. Aniridia is an autosomal dominant disorder caused by haploinsufficiency of the highly conserved transcriptional factor PAX6 (Paired-box gene 6), which is involved in the morphogenesis and maintenance of ocular structures [2]. Patients with *PAX6*-related abnormalities show overlapping presentations of different anterior and posterior segment anomalies [1,3,4]. Patients carrying *PAX6* pathogenic variants usually present with iris defects to varying degrees ranging from complete absence to a normal iris with minor abnormalities [1,3,4], but foveal hypoplasia, responsible for congenital low vision, is even more frequent [3]. Iris and foveal hypoplasia are often accompanied by other ocular manifestations, such as early-onset glaucoma, cataract, and limbal dysfunction, among others, leading to a loss of visual acuity throughout life [1,3,4].

The *PAX6* gene consists of 14 exons, including an alternative exon between exon 5 and 6, hereafter, called exon 5a. Three promoters, P0, P1, and Pα, control *PAX6* transcription [5]. On the one hand, P0 produces a transcript (RefSeq NM_000280) encoding a canonical 422 amino acid PAX6 protein containing two DNA binding domains, the DNA-binding paired domain (PD) and homeodomain (HD) and a proline-serine-threonine (PST)-enriched transactivation domain that regulates the transcription of target genes. On the other hand, the inclusion of exon 5a is controlled by P1 and generates a larger protein isoform PAX6-5a of 436 amino acids (Ref Seq NM_001604) [5,6]. This alternative exonic inclusion leads to PD disruption, mediating the recognition of different DNA targets. Finally, the internal promoter Pα regulates the expression of PD-truncated proteins (PAX6ΔPD), which can bind to DNA only through HD [5,6,7]. While the function of PAX6ΔPD remains unknown, its overexpression has been described to alter corneal and lens development, leading to microphthalmia in mouse models [8].

So far, more than 700 single-nucleotide variants (SNVs), small indels, and copy-number variants (CNVs) affecting *PAX6* have been reported in mutational databases (LOVD and HGMD databases, last accession November 2022) and/or in the literature [9]. Most patients with aniridia carry loss-of-function (LOF) variants that introduce a premature termination codon (PTC). Consequently, mutant transcripts are thought to be targeted by the nonsense-mediated mRNA decay (NMD) system [10,11]. Additionally, there is increasing evidence that defects in *PAX6* pre-mRNA splicing represent a major cause of aniridia, with more than 100 described splicing variants accounting for up to 15% of disease-causing variants [7]. Most of them affect conserved nucleotides at exon–intron boundaries, i.e., canonical acceptor or donor splicing sites (3′SS and 5′SS, respectively). Still, others involve non-canonical splicing sites (NCSS) or splicing regulatory elements (SRE) [12,13].

In recent years, the advent of next-generation sequencing (NGS) has allowed the screening of the entire *PAX6 locus*, including the UTRs and introns, leading to the identification of a substantial number of non-coding variants in *PAX6*-negative patients [13,14]. Furthermore, most of them negatively impact splicing by creating or enhancing exonic and deep-intronic NCSS [12,13,14]. Variants in NCSS are especially relevant for the major mutational hotspot, the *PAX6* exon 6, which encodes the PD [7]. In this exon, a complex scenario for alternative splicing has been envisaged. This is mediated by a plethora of exonic and intronic cryptic 5′SS and 3′SS [12,13,15]. However, the full splicing regulation in *PAX6* exon 6 remains to be determined.

Despite the availability of new in silico splice prediction tools, their ability to predict cryptic NCCS and deep-intronic variants (DIVs) is still limited [16]. Thus, the precise implication of variants affecting NCSS will remain difficult to establish unless appropriate RNA expression studies are performed for splicing assessment [17]. However, direct RNA analysis is not feasible in patients with aniridia, given the specific expression of *PAX6* in non-accessible tissues. Alternatively, owing to illegitimate *PAX6* expression in Epstein–Barr-immortalized lymphocytes, the spliceogenic effects of some *PAX6* variants have been characterized using patient-derived lymphocyte cell lines (LCLs) [13,15,18,19].

To overcome these shortcomings in the feasibility of RNA analyses, in vitro minigene-based assays have become widely available and are easy-to-perform tools for the functional assessment of splicing variants on pre-mRNA maturation [17]. Minigenes are plasmid-based constructions consisting of a simplified part of a gene with the exon of interest and its closest flanking intronic regions [20]. Accordingly, minigene-based splice assays have been used in recent years to experimentally confirm different exonic and near-exon spliceogenic variants in *PAX6* [12,13,14,21,22,23,24]. This approach has provided insight into the pathogenic mechanisms behind a priori variants of unknown significance (VUS); thus, a definite genetic diagnosis was made, solving part of the “missing heritability” in aniridia [13,14].

Herein, we aimed to functionally characterize four DIVs in *PAX6* identified in patients with aniridia. For that, we assessed their spliceogenic effect by combining multi-exonic minigene assays and nanopore-based long-read sequencing (LRS) for accurate quantification and determination of full-length *PAX6* alternative transcripts. Our findings confirm the implication of two DIVs in intron 6 of *PAX6* that cause aberrant mRNA pre-processing through several new and cryptic intronic 5′SS and 3′SS, leading to exonization events mediated by pseudoexon (PSE) inclusions and intronic retentions.

## 2. Results

### 2.1. Four Deep-Intronic Variants Detected in PAX6

Following comprehensive sequencing of the full-length *PAX6* gene and its regulatory elements using targeted NGS approaches, we identified four DIVs in patients with congenital aniridia with no other potentially pathogenic SNVs or CNVs identified. These DIVs were absent or very rare in population databases, were predicted to have a potential splicing effect by in silico splicing predictor tools, and following ACMG criteria, were considered as VUS at the time of identification, as summarized in Table 1. On the one hand, two DIVs were identified in intron 6, c.357+136G>A and c.357+334G>A. These variants were previously described in two French probands with congenital aniridia [14]. The first variant, c.357+136G>A, was detected in a sporadic case, in which segregation analysis suggested a de novo event as neither parent was a carrier. This variant was predicted to create a new strong 3′SS (MaxEnt score 9.9 out of 12) at a deep-intronic position c.357+138 of intron 6, as suggested by seven splicing predictors (Table 1). ESEFinder also predicted the creation of novel exonic splicing element (ESE) motifs in this region.

The second variant, c.357+334G>A, was identified in the index case of a two-generation family with five individuals suffering from congenital aniridia. A segregation analysis was not done due to the lack of DNA samples from affected family members. This variant was also predicted to create or strengthen a cryptic deep-intronic 5′SS at c.357+331 (MaxEnt score 7.3 out of 12), as revealed by 6 out of 7 predictors (Table 1).

On the other hand, two DIVs were identified in intron 11, c.1032+170A>T and c.1033-275A>C. The first variant, c.1032+170A>T, was detected in a sporadic Spanish patient with iris hypoplasia, Axenfeld–Rieger anomaly, and congenital glaucoma with no family history. No causal variants in *FOXC1*, *PITX2,* or other genes involved in this phenotypic presentation were detected during gene panel screening. This novel variant appeared to potentially create a new deep-intronic 3′SS in c.1032+168 (MaxEnt score 6.8 out of 12), as revealed by two predictors (Table 1). In addition, a significant alteration in the ratio of ESE to exonic splicing silencers (ESS) was also predicted (Table 1). The variant c.1033-275A>C was previously reported in a sporadic French patient with a complex phenotype of aniridia, microphthalmia, and microspherophakia [14]. Two in silico tools (SFF and MaxtEnt) predicted that this change might potentially create or slightly strengthen a deep-intronic weak cryptic 3′SS in c.1033-262 (MaxEnt score 2.8 out of 12) (Table 1). The inheritance remains unknown for the latter two variants due to the unavailability of parental samples for segregation analysis.

### 2.2. Long-Read Sequencing for the Analysis of Complex Splicing Events

**Figure 1:**
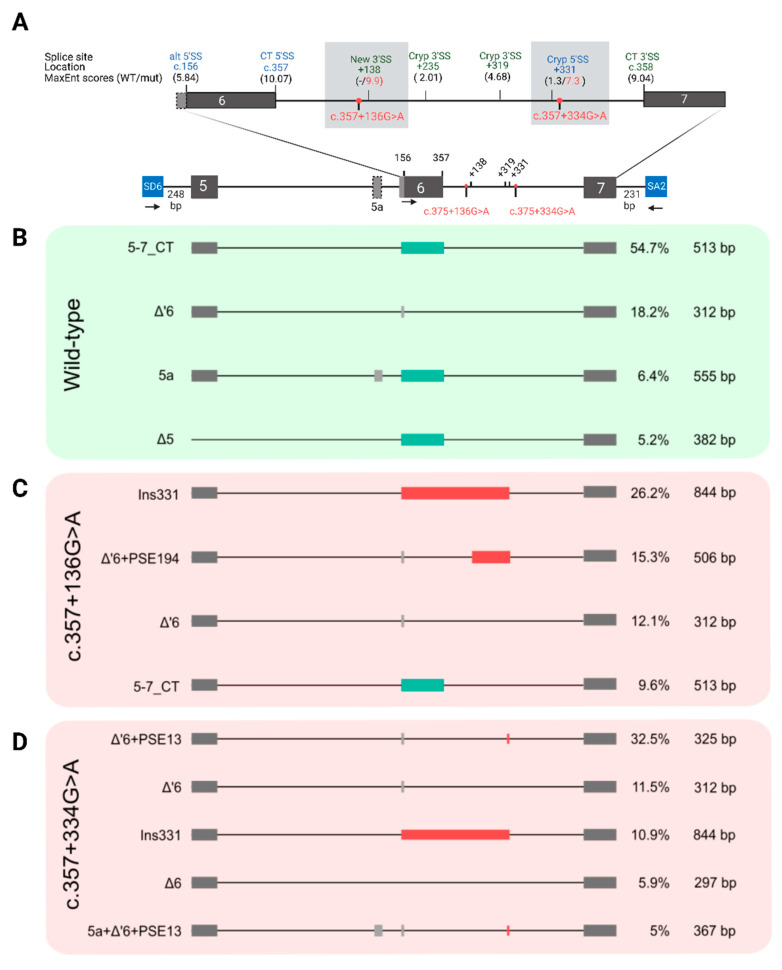
Splicing outcomes for two spliceogenic deep-intronic variants located in intron 6 of *PAX6* using in vitro minigene assays. (**A**) Schematic representation of the multi-exonic minigene construction generated from an exon trapping pSLP3 vector (blue boxes) containing exons 5 to 7 of *PAX6*. The alternative 5 exon (5a) and the partially skipped exon 6 (∆’6) are shown with dashed lines. Localization of the deep-intronic variants c.357+136G>A and c.357+334G>A (red dot) on intron 6 and the primers (arrowheads) used for RT-PCR amplification for long-read sequencing (SD6-F and SA2-R) and semi-quantitative PCR (PAX6-F and SA2-R) are shown. The natural donor (5′SS, blue) and acceptor (3′SS, green) splicing sites of exons 6 and 7 are represented as well as different alternative (alt) and predicted cryptic (cryp) splicing sites, indicating their splice prediction scores from the MaxEnt tool (WT and mutant scores are colored in black and red, respectively). (**B**–**D**) Isoform patterns, their relative abundances (in percentages), and isoform sizes (in base pairs) for the wild-type and the two mutated constructions obtained by long-read sequencing of RT-PCR fragments. Only isoforms with values above a threshold of 5% are shown. (**B**) Wild-type construction yielded three predominant isoforms corresponding to the canonical transcript (5–7_CT), including exons 5, 6, and 7, and two alternative isoforms, a partially skipped exon 6 (∆’6) and the alternative 5 exon (5a). (**C**,**D**) For the mutant minigenes, a mixture of aberrant splice amplicons (in red) was obtained, including a shared intronic retention of 331-nt (Ins331) and the inclusion of pseudoexons (PSEs) of 194-nt (PSE194) and 13-nt (PSE13) (not shown) for c.357+136G>A and 13-nt (PSE13) and 97-nt (PSE97) (not shown) for c.357+334G>A. SS: splicing site. Cryp: cryptic. CT: canonical transcript. PSE: pseudoexon insertion. Ins: insertion.

**Figure 2:**
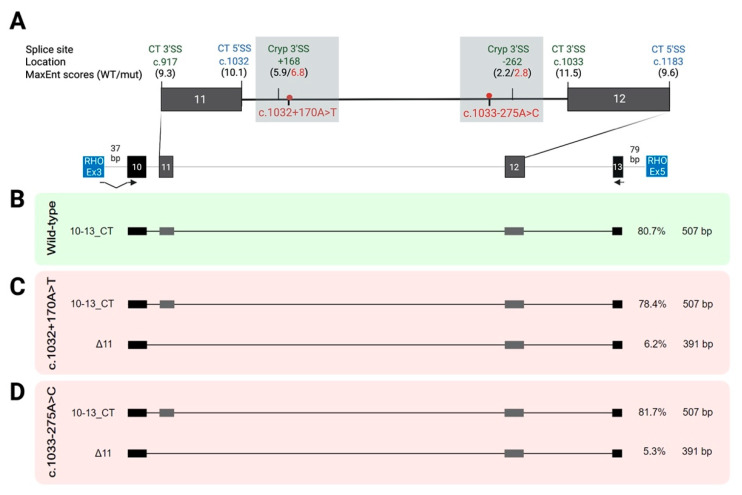
Splicing outcomes for two spliceogenic deep-intronic variants located in intron 11 of *PAX6* using in vitro minigene assays. (**A**) Schematic representation of the multi-exonic minigene construction generated from an exon trapping pCI-NEO-RHO-exon3,5/DEST (blue boxes) containing exons 10 to 13 of *PAX6*. Localization of the deep-intronic c.1032+170A>T and c.1033-275A>C (red dots) on intron 11 and primers (arrowheads) used for RT-PCR amplification for LRS and semi-quantitative PCR are shown (Rho_PAX6-F and PAX6-R). The natural donor (5′SS, blue) and acceptor (3′SS, green) splicing sites of exons 11 and 12 are presented as well as the predicted cryptic (cryp) splicing sites, indicating their splice prediction scores obtained from the MaxEnt tool (WT and mutant scores are colored in black and red, respectively). (**B**–**D**) Isoform patterns and their relative abundances (in percentages) and isoform sizes (in base pairs) for the wild-type and the two mutated constructions obtained by long-read sequencing of RT-PCR fragments. Only isoforms with values above a threshold of 5% are shown. (**B**) The wild-type minigene yielded a unique predominant isoform corresponding to the canonical transcript (10–13_CT), including exons 10, 11, 12, and 13. (**C**,**D**) For the mutant minigenes, two major isoforms are present, canonical transcript (10–13_CT) and a minor isoform with deleted exon 11 (Δ11). SS: splicing site. Cryp: cryptic. CT: canonical transcript. RHO: rhodopsin.

To functionally assess the suspected repercussion of these DIVs on splicing, we carried out in vitro minigene splicing assays owing to the unavailability of patient-derived LCLs. For that, two multi-exonic minigene constructions encompassing exons 5 to 7 (PAX6_5–7 WT minigene) and exons 10 to 13 (PAX6_10–13 WT minigene) of *PAX6* were generated to analyze DIVs in introns 6 and 11, respectively (Figure 1A and Figure 2A).

**Table 1:** Summary of the identified deep-intronic variants in *PAX6*, in silico predictions, and the main in vitro outcomes using multi-exonic minigenes.

Variant	c.357+136G>A	c.357+334G>A	c.1032+170A>T	c.1033-275A>C
Patient	SG102229	SG132488	V-0238	ADN130014
Family history	Sporadic	Familial	Sporadic	Sporadic
Intron	6	6	11	11
Inheritance	De novo	NA	NA	NA
Phenotype	Isolated aniridia	Isolated aniridia	Anterior segment dysgenesis, iris hypoplasia, congenital glaucoma	Aniridia microphthalmia microspherophakia
Allele frequency gnomAD	0	0	0.000006982	0.00156
Reference	Plaisancie, 2018 [14]	Plaisancie, 2018 [14]	This study	Plaisancie, 2018 [14]
In silico consequence	Creating new 3′SS	Strengthening cryptic 5′SS	Strengthening cryptic 3′SS	Creating/strengthening cryptic 3′SS
Position splicing site	c.357+138	c.357+331	c.1032+168	c.1033-262
SSF [0–100]	−/83.1	−/71.8	=71.4	−/71
MaxEnt [0–12]	−/9.9	1.3/7.3	5.9/6.8	2.2/2.8
NNSPLICE [0,1]	−/0.8	−/0.8	-	-
GeneSplicer [0–15]	−/7	-	-	-
HSF [0–100]	−/83.2	75.7/76.8	-	=73.4
VarSEAK	−/+49.7%	−20.5%/+22.9%	-	-
SpliceAI [0,1]	0.39	0.46	0	0
ESE Finder	Creating new ESE motifs		Significant alteration of ESE/ESS motifs ratio	
In vitro outcomes	Aberrant splicing (Ins331, PSE194, PSE13)	Aberrant splicing (Ins331, PSE97, PSE13)	WT-like splicing pattern	WT-like splicing pattern
ACMG classification	4 (PM2, PP3, PP4, PS2, PS3)	4 (PM2, PP3, PP4, PS3)	3 (PM2, PP3, BS3)	1 (BS1, BP6, BP7, BS3)

3′SS, acceptor splicing site; 5′SS, donor splicing site; ACMG, American College of Medical Genetics; Ins331: elongated isoform for exon 6 retaining 331 nucleotides from intron 6, PSE; pseudoexons in intron 6 indicating the size in nucleotides; ESE: exonic splicing enhancer; ESS: exonic splicing silencer; NA, no data available; WT, wild-type.

Given the known complexity of alternative splicing for exons 5 and 6 [13], we used a novel approach based on multiplexed nanopore-based long-read sequencing to facilitate the analysis and quantification of the mixture of full-length transcripts derived from minigenes. LRS revealed a high number of splicing events for the PAX6_5–7 WT construction, which yielded four isoforms with an abundance above 5%, as shown in Figure 1B and schematically depicted in Figure S1A. Three major isoforms accounted for almost 80% of the transcripts detected by LRS: (i) an isoform corresponding to the canonical transcript 5–7_CT (RefSeq NM_000280); (ii) an isoform of the partially skipped exon 6 (∆’6) that includes only 15 bp of the exon due to the use of an alternative 5′SS at position c.156; and (iii) an isoform corresponding to the canonical RefSeq transcript NM_001604 that includes the alternative exon 5a (5a_CT). These isoforms correspond to previously reported alternative splicing events [13,15]. In addition, a fourth isoform in which exon 5 is skipped (∆5) was detected, accounting for 5.2% of transcripts. Other minor isoforms were detected but with abundance levels of below 5% (Figure S2A).

### 2.3. Two Deep-Intronic Variants in Intron 6 Lead to Aberrant Splicing

Minigene splice assays of the c.357+136G>A and c.357+334G>A variants, combined with the LRS analysis, revealed a very complex isoform pattern compared to WT due to the presence of a variety of aberrant splicing events, leading to the exonization of intron 6 (Figure 1C,D).

First, the variant c.357+136G>A induced three non-canonical splicing events with the retention of the first 331 intronic nucleotides (Ins331) and the inclusion of two pseudoexons of 197 and 13 bp, respectively (Figure 1C). The major aberrant splicing transcript (26.2%) was Ins331, in which exon 6 is elongated. Moreover, this retention was present in two other minor isoforms with values below the 5% threshold (Figure S2B), accompanied by the alternative exon 5a (4.9%) or by the complete skipping of exon 5 (1.1%). Thus, this 331 nt-intronic retention accounted for 32.2% of all transcripts. The second most abundant splicing event corresponded to the inclusion of a 194-nt pseudoexon (PSE194) (15.3%) between the partially skipped exon 6 (∆’6) and exon 7. In addition, this aberrant PSE inclusion was found in two minor transcripts (Figure S2B), between full-sized exons 6 and 7 or accompanied by the alternative isoform 5a-∆’6 (3.6% and 2.9%, respectively). Thus, PSE194 was present in 21.8% of all transcripts. Furthermore, a 13-nt-PSE (PSE13) was induced by this variant, and this was mainly accompanied by ∆’6 in a minor isoform accounting for 4.6% of all transcripts. Lastly, the natural splicing isoforms 5–7_CT and ∆’6 were also detected (9.6 and 12.1%, respectively), but both showed a decrease with respect to the WT construct. Sanger sequencing and a semi-quantitative capillary analysis (Figure S3) were performed to validate the LRS findings.

The minigene assay for the variant c.357+334G>A also revealed an abnormal splicing pattern with three aberrant splicing events: intronic 331-nt retention and two pseudoexon inclusions of 97 and 13 nt, respectively (Figure 1D). The major splicing isoform with an abundance of 32.5% carried PSE13, and this was mainly accompanied by partially skipped exon 6. PSE13 was also found in other minor isoforms (Figure S2C) and was mainly accompanied by the 5a+∆’6 and 5–7_CT events (5% and 4.3%, respectively), leading to an absolute abundance in 44.4% of all transcripts. A second major abnormal event was the 331bp-elongated exon 6 (10.9%). Two other less abundant aberrant events were also detected (Figure S2C), full-skipped exon 6 (∆6) (5.9%) and a 97-nt pseudoexon (PSE97) derived from intron 6 that was included in the natural isoforms 5–7_CT and ∆’6 (1.6% and 3.8%, respectively). Finally, regular splicing events were detected, the canonical 5–7_CT and ∆’6 isoforms (4.9% and 11.5%, respectively), whereas the alternative 5a isoform was completely missed (Figure S2C).

Given these findings, we were able to correlate the in silico predictions with the observed in vitro outcomes for these two deep-intronic variants in intron 6, which are schematically represented in Figure S3A. In the case of variant c.357+136G>A, the minigene analysis confirmed that the predicted novel deep-intronic 3′SS at position c.357+138 was involved in forming PSE194. Moreover, a cryptic 3′SS at position c.357+319 was used to create PSE13. During these aberrant splicing events, a predicted cryptic deep-intronic 5′SS at c.357+331 was used as the donor site in these pseudoexons. In the mutant construct, this cryptic 5′SS was also used preferentially to the canonical 5′SS, leading to the 331-nt retention of intron 6. For variant c.357+334G>A, a similar effect was observed in the minigene analysis, confirming the strengthening of this cryptic deep-intronic 5′SS. Its use directly led to the elongation above exon 6, but it was also involved in the creation of two pseudoexons, PSE13 and PSE97. For that, two cryptic deep-intronic 3′SS, located at positions c.357+319 and c.357+235, respectively, were used. Notably, the three aberrant PSEs arising from both DIVs were found to overlap, as they all use the same 5′SS located at position c.357+331. Therefore, the size of each pseudoexon is related to the position at intron 6 of the alternative cryptic 3′SS used.

All of these aberrant splicing events could induce a variety of frameshifts, such as p.Val120Asnfs*17, p.Val120Glyfs*11, p.Val120Glyfs*48, and p.Ile123Leufs*28 in the cases of PSE13, PSE97, PSE194, and Ins331, respectively. Therefore, DIVs in intron 6 could result in truncated proteins that would potentially be degraded by NMD activation, leading to *PAX6* haploinsufficiency.

### 2.4. Two Deep-Intronic Variants in Intron 11 Did Not Affect Pre-mRNA Maturation

In vitro analysis of the deep-intronic variants c.1032+170A>T and c.1033-275A>C, located in intron 11, was performed using a minigene containing the entire region between exons 10 to 13, and the derived RNA isoforms were quantified by LRS (Figure 2 and Figure S4A) and further validated by semi-quantitative electropherograms (Figure S5).

From the WT PAX6_10–13 minigene originated a major canonical isoform containing all four correctly spliced exons (Figure 2B) as well as three minor alternative transcripts (Figures S1B and S4A): (i) an isoform with exon 11 skipped; (ii) an isoform with exon 12 skipped, and (iii) an isoform with both exons 11 and 12 skipped. These isoforms accounted for 80.7%, 4.8%, 4.6%, and 4.5% of the total, respectively. A similar isoform pattern was also obtained for the two mutant constructs (Figure 2C,D and Figure S4B,C). Therefore, these DIVs did not disturb the normal splicing process.

## 3. Discussion

Exome sequencing approaches enable the successful study of coding regions and canonical splice sites in the human genome. However, in many patients, the genetic cause of their disease remains unclear [25]. One of the reasons for these elusive causative variants is that they are located in non-coding regions, which are missed during coding-region-targeted sequencing. Nevertheless, the use of custom panels designed to cover non-coding regions and regulatory elements of target genes and the growing implementation of WGS have enabled the detection of non-coding variants, such as DIVs [25]. Despite this, interpreting the pathogenic implications of these variations is still challenging. One of the underlying pathogenic mechanisms is their implications in pre-RNA processing. They could lead to aberrant mRNA transcripts by altering canonical or cryptic 5′/3′SS or regulatory element binding sites and creating new ones, which could cause a vast variety of splicing events, such as total or partial exon skipping, intron retention, or PSE inclusion [16]. The causality of potential spliceogenic variants in NCSS is highly challenging to verify unless functional validation is performed.

Here, we aimed to functionally characterize the only three DIVs reported to date in *PAX6* (to the best of our knowledge) and one newly identified variant. In silico studies of these DIVs predicted the implication of deep-intronic sites. In silico predictions are not sufficient evidence to establish actual effects on pre-mRNA processing [16]. This is the case for three DIVs identified several years ago in patients with aniridia [14], which have remained inconclusive to date.

Splicing analysis is highly recommended to gain insight into the pathogenic effects of non-coding splicing variants and help with their clinical interpretation. Considering that *PAX6* shows restricted expression in non-clinical tissues, minigene assays represent an alternative way to assess pre-mRNA expression [26]. In the case of deep-intronic variant assessment, multi-exonic minigene constructions help to preserve the genomic context thanks to the inclusion of upstream and downstream exon–intron recognition signals or intronic regulatory elements that might influence the splicing process [27,28,29].

The obtained outcomes from the multi-exon *PAX6* minigene assays improved the clinical classification of the four DIVs studied herein by incorporating the ACMG PS3/BS3 criteria during the evaluation. The two DIVs in intron 6 were upgraded to likely pathogenic (class 4), as our findings confirmed that both variants induce the abnormal exonization of intron 6. All aberrant isoforms would generate potentially truncated proteins, possibly leading to NMD activation. Another important consequence observed was a significant reduction in the canonical *PAX6* compared to the WT construct, contributing to *PAX6* haploinsufficiency.

Regarding the aberrant splicing of intron 6, seven in silico prediction tools predicted the involvement of two intronic sites. However, a more complex scenario was observed for the underlying isoform patterns of both DIVs, bringing to light the actual involvement of four intronic splicing sites (Figure 1A). First, the alternative use of a cryptic 5′SS at intronic position +331 is a crucial element involved in the formation of all exonization events in intron 6 (Figure S3A). It is used alternatively to the natural 5′SS in the presence of both DIVs. The direct common consequence is the insertion of intronic sequences, leading to a 331-nt elongation of exon 6. In the case of c.357+334G>A, the presence of this retention confirms the predicted direct strengthening of this cryptic 5′SS. This aberrant isoform is most frequently induced by c.357+136G>A, indicating that this variant may activate splicing enhancers, as also predicted in silico. We could hypothesize whether these ESEs could enhance the use of this distant cryptic 5′SS. Interestingly, similar intronic retention is part of a minor *PAX6* transcript reported in UCSC and Ensembl databases which arises from the alternative promoter Pα in intron 4 [5,6,7]. Further studies should be performed to evaluate whether this intronic retention is present in patients carrying c.357+136G>A and healthy controls, as well as to study its implication in the alternative splicing of *PAX6* during ocular development.

Furthermore, three intronic 3′SS are involved in the complex splicing patterns for both DIVs in intron 6. As predicted, a novel 3′SS is created at position c.357+138 as a direct consequence of the nucleotidic change c.357+136G>A, leading to the introduction of a 194-nt intronic PSE. Additionally, a cryptic 3′SS at intronic position +319 was found to be involved in the formation of a 13-nt PSE. In the presence of c.357+334G>A, two cryptic 3′SS at intronic positions +235 and 319 were also unexpectedly found to be involved in the creation of two PSEs of 97 and 13-nt, respectively.

Regarding the DIVs in intron 11, c.1032+170A>T and c.1033-275A>C, which were detected in patients suffering from *PAX6*-related phenotypes, some in silico tools suggested the potential creation of two novel 3′SS located at positions c.1032+168 and c.1033-262, respectively. Although LRS revealed a more complex pattern for the splicing of the region studied, neither DIVs altered the correct splicing observed in the WT construct. Both variants are additional examples of conflicting variants in which the clinical interpretation changed over time thanks to accumulated knowledge from the literature, population and clinical databases, and new functional data. When reported in 2018, we considered the variant c. 1033-275A> to be VUS, given its potential splicing effect, but a recent ClinVar entry classified it as benign. In addition, our functional study supports its non-pathogenicity, and the ACMG BS3 criterion can now also be used during clinical classification, obtaining a definite class 1 score. For c.1032+170A>T, functional findings did not support its pathogenicity in terms of splicing, but it is still considered VUS, even taking into account the BS3 criterion. The lack of segregation data for this variant prevents a conclusive clinical interpretation being reached. Our findings reflect the difficulties involved in interpreting splicing variants, highlighting that caution must be taken, especially in the clinical context.

Splicing analysis can be challenging when dealing with complex isoform patterns, as is the case for variants affecting *PAX6* exons 5 to 6, where alternative splicing generates multiple partially redundant isoforms [13]. Minigene analysis of this region using gel-based electrophoresis and Sanger sequencing is laborious, and it is difficult to establish the complete outlook of splicing events, so the actual isoform landscape may remain masked. Alternatively, LRS can quickly identify and quantify complex mixtures of full-length transcripts [30,31]. Here, we successfully applied LRS to facilitate the splicing analysis of this challenging gene. The proposed approach combines nanopore-based LRS of minigene-derived RT-PCR amplicons. In addition, this approach allowed the development of a new custom bioinformatic tool, VIsoQLR, which defines de novo splice junctions and directly quantifies the transcript abundance [32], allowing the study of alternative splicing methods and differential isoform expression. A second advantage of LRS applied to splicing analysis is the ability to discriminate among isoforms with minor size differences without the need of laborious cloning for Sanger sequencing. This is especially relevant when discerning between the canonical *PAX6* transcripts of naturally spliced alternative isoforms (5a and ∆’6) or the inclusion of a 13-nt PSE, which involves the skipping or inclusion of only a few nucleotides. Lastly, LRS allows full-sized transcripts with exact lineal exon structures to be obtained, unlike other short-read RNA-seq strategies. Therefore, LRS is a new technique of choice that can be used to decipher the precise and complete isoform patterns associated with potential spliceogenic variants.

Despite the clear advantages and usefulness of in vitro minigene splicing analysis, artificial approaches do not always mimic actual mRNA processes in affected tissues. Caution should be exercised, especially when concluding the absence of splicing consequences. Since splicing is regulated in a tissue-dependent manner by specific intronic and exonic SREs, splicing outcomes may be biased by the cellular context. In this sense, immortalized cell lines may behave differentially with respect to patient tissues, so different outcomes may be obtained depending on the nature of the tissue from which the RNA is derived [33]. Alternatively, adult somatic cell reprogramming techniques circumvent the inaccessibility of human ocular tissues by generating patient-derived induced pluripotent stem cells (iPSC) [34,35,36]. Although only two iPSC lines carrying *PAX6* variants have been generated to date [37,38], the growing ability of differentiation methods to develop a variety of iPSC-derived ocular organoids that can mimic the human optic cup, retina, cornea or lentoid bodies in vivo allows the study of oculogenesis in the early stages [35,39,40,41]. Therefore, the use of organoids as disease models opens the possibility of assessing splicing variants under more physiological conditions [42], as well as deepening the involvement of *PAX6* alternative splicing in the development of different ocular structures.

In conclusion, our work supports the idea that cryptic splicing sites in intron 6 of *PAX6* contribute to mis-splicing in congenital aniridia, expanding the knowledge on mutational mechanisms associated with *PAX6*. Determination of the underlying splicing mechanism, which implicates a shared cryptic intronic splicing site, will open new opportunities for therapeutic approaches based on antisense oligonucleotide-mediated exon skipping to suppress defective splicing and rescue canonical transcripts. Our study highlights the importance of combining thorough screening of non-coding regions with functional assays to decipher the missing heritability in congenital aniridia. A splicing analysis of four NCSSs in *PAX6*, previously considered variants of unknown significance, aided in their re-classification and, thus, allowed for conclusive genetic diagnosis and counselling.

## 4. Materials and Methods

### 4.1. Patient Recruitment and Genetic Analysis

Three of the patients discussed herein were carriers of the variants c.357+136G>A, c.357+334G>A, and c.1033-275A>C who had been clinically diagnosed with congenital aniridia in the Hôpital Purpan (Toulouse, France). Genetic screening of *PAX6* was performed by a gene-locus NGS approach, as previously described [14]. The patient carrying the variant c.1032+170A>T was recruited and diagnosed at the Fundación Jiménez Díaz University Hospital (Madrid, Spain), and *PAX6* screening was conducted by NGS with a panel of genes implicated in congenital ocular malformations, as previously described [13].

### 4.2. In Silico Analysis

The potential pathogenicity of the DIV in splicing was assessed with the following 7 algorithms: the Human Splicing Finder [43], GeneSplicer, MaxEntScan, NNSPLICE, Splice Site Finder with Alamut visual software version 2.11 (Interactive software, Rouen, France), VarSEAK (https://varseak.bio/, accessed on 15 October 2022), and SpliceAI [44]. These were used to assess the strength of canonical and potential new/cryptic splicing sites. The ESEfinder from Alamut was used to identify putative SRE-associated sites. Additional pathogenic predictors, including the CADD and DANN tools, were used. The minor allele frequency (MAF) was defined using the gnomAD database.

### 4.3. Minigene Splicing Assays

To assess the pathogenic impact on the splicing of these variants, an in vitro approach based on multi-exonic minigenes assays was designed. To that aim, two multi-exonic minigenes covering exons 5 to 7 and 10 to 13, respectively, were generated.

On the one hand, the region of interest spanning exons 5 to 7 of the RefSeq *PAX6* isoform NM_000280.4 and around 200-nt flanking intronic sequences were amplified from a healthy control’s DNA using FastStartTaq DNA Polymerase (Roche Diagnostics, Basel, Switzerland) and specific forward and reverse primers with XhoI-BamHI or EcoRV-VspI tails, respectively, to allow subsequent cloning. A 1% agarose gel was used to check that a single-band PCR product was obtained. The amplicon was firstly subcloned into a pCR2.1-TOPO-TA Vector using the TOPO TA Cloning Kit (Invitrogen, Thermo Fisher Scientific, Waltham, MA, USA) and then cloned into the exon trapping expression pSPL3 vector by a classical restriction digestion/ligation strategy using BamHI and NotI (Thermo Fisher Scientific) and T4 DNA ligase (Roche Diagnostics). The final wild-type (WT) minigene construction included 248 bp of flanking intron 4, exon 5, the complete intron 5, including an alternative exon herein referred to as exon 5 alternative (5a), exon 6, the entire intron 6, exon 7, and 231 bp of flanking intron 7 (Figure 1A).

On the other hand, a minigene composed of exons 10 to 13 of *PAX6* was generated. To this aim, the region of interest was amplified from commercial DNA using a proofreading Platinum SuperFi DNA Polymerase (Invitrogen) and checked by gel electrophoresis. The forward primer used is located at intron 9 (37 bp from the beginning of exon 10), and the reverse primer is located in the 3′UTR region (79 bp from the end of exon 13). A tail containing the recombination sites for subsequent cloning was added to the 5′ end of both primers. The final sequence of the primers is shown in Table S1. The PCR product was then purified with PEG following the purification protocol of Gateway technology (Invitrogen). The cloning process used to obtain the minigene was carried out in two steps. First, around 100 nanograms of purified amplicon was cloned into the pDONR221 donor vector using the Gateway BP Clonase II Enzyme mix (Invitrogen). Second, to transfer the fragment of interest from pDONR221 to pCI-NEO-RHOexon3,5/DEST exon-trapping expression vector [33], 150 ng of the pDONR221construct, including exons 10 to 13 of *PAX6*, and the Gateway LR Clonase II Enzyme mix (Invitrogen) was used for cloning. The resulting WT multi-exonic minigene pRHO_*PAX6*_ex10–13 was composed of the whole sequence between exon 10 and 13 (including complete introns) and by 37 and 79 bp of the flanking regions at each end (Figure 2A).

Sanger sequencing was then performed to verify the correct insertion of the *PAX6* sequences compared to the human reference genome GRCh37/hg19. Sequence analysis of WT constructs revealed intronic non-pathogenic polymorphisms located at intron 6, for which an in silico study did not reveal any potential effects on splicing.

Mutant minigenes were obtained for the DIVs by site-directed mutagenesis with the NZYMutagenesis Kit (NZYTech, Lisbon, Portugal). The primers used for genomic amplification and site-directed mutagenesis are listed in Table S1. Two micrograms of WT and mutant constructions were transfected in HEK-293T cells using the Jet-Pei reagent (Polyplus, Illkirch-Graffenstaden, France), as previously described [13,14].

### 4.4. RT-PCR

Total RNA from transfected HEK-293T cells was isolated using the TRIzol reagent and Phasemaker tubes (Invitrogen). Reverse transcription of 2.5 µg of RNA was performed using random hexamers and the Superscript IV First-Strand Synthesis System (Invitrogen) in accordance with the manufacturer’s conditions. Further PCR was performed using a vector-specific primer pair (Table S1) to amplify the full-length splicing isoforms derived from the minigene. The PCR conditions used were as follows: 2 µL of cDNA, 2 µL of 10X PCR FastStart Buffer (Roche Diagnostics), 0.8 µL of 10 µM primer, 3.2 µL of 1.25 mM dNTP, and 0.2 µL of 5 U/µL FastStart Taq DNA Polymerase (Roche Diagnostics) per 20 µL/mix. The cycling conditions were 95 °C for 5 min of initial denaturation, 95 °C for 30 s, 60 °C for 30 s, 72 °C for 50 s, according to the fragment size (35 cycles), and 72 °C for 7 min.

### 4.5. Multiplexed Targeted Nanopore Sequencing

To fully sequence and quantify each isoform, LRS of the mRNA splicing patterns for the WT and the mutant minigenes was conducted on a MinION device (Oxford Nanopore Technologies, ONT, Oxford, UK) using a multiplexed assay.

Library preparation was carried out using the SQK-LSK109 sequencing kit (ONT) in accordance with the recommended protocol “Native barcoding amplicons” (ONT, version NBA_9093_v109_revF_12Nov2019). Briefly, PCR-derived amplicons were fluorometrically quantified using the Qubit dsDNA HS Assay kit (Invitrogen), and a total of 100–200 fmol of PCR product was used for the barcoding reaction. The WT isoform size (N) was used to calculate the quantity in fmol needed, according to the following formula: ng = (x fmol)*(N)*(660 fg/fmol)*(1 ng/10^6^ fg). A first step of 5′ end-repair and 3′ dA tailing was conducted using the NEBNext Ultra II End Repair/dA-Tailing Module (New England BioLabs, NEB, Ipswich, MA, USA). Secondly, end-prepped DNA was barcoding using the Native barcoding expansion kit (EXP-NBD104, ONT) and NEB Blunt/TA Ligase Master Mix (NEB). Thirdly, after fluorometric quantification using the Qubit dsDNA HS Assay kit (Invitrogen), barcoded samples were pooled equimolarly (a 150-fmol total). Lastly, adapter ligation to the pooled-barcoded samples and clean-up were conducted using the NEB Next Quick Ligation Module and the ONT SQK-LSK109 Ligation Sequencing kit. The final adapted-ligated DNA library was fluorometrically quantified, and 40 fmol was loaded into SpotOn Flow Cell R9 Version using the ONT Flow Cell Priming kit. The 12-plex library was further sequenced into a MinION Mk1B device using MinKNOW software (ONT) with default parameters until all samples had overcome a threshold of 5000 reads.

### 4.6. Quantitative Analysis of Splicing Events

The complete LRS analysis was performed using VisoQLR (https://github.com/TBLabFJD/VIsoQLR, accessed on 31 October 2022) [32]. First, reads were aligned to the reference sequence using the GMAP plugin [45] with VisoQLR generating GFF3 files. These files were then used as input into the same tool for the isoform analysis and quantification. The “read threshold” for the automatic detection of exon coordinates was set at 3% for all experiments, and manual curation of detected exon coordinates was performed on reads from c.1032+170A>T and c.1033-275A>C.

### 4.7. Validation of Splicing Events

To validate the LRS results, the splicing events were analyzed by RT-PCR followed by Sanger sequencing and semi-quantitative quantification. Two microliters of cDNA was used as template for semi-quantitative PCR and Sanger sequencing under the conditions mentioned above. The sequences and locations of primer combinations used to amplify the obtained isoforms are listed in Table S1 and shown in Figure 1A and Figure 2A. For semi-quantitative PCR, HEX-labelled reverse primers were used.

For Sanger sequencing, PCR products were run on a 2% agarose gel. Bands were then excised and purified with the NucleoSpin Gel and PCR Clean-up kit (Macherey-Nagel, Düren, Germany) and sequenced. For semi-quantitative PCR, labelled-PCR products were run together with ROX1000 (Asuragen, Austin, TX, USA) or LIZ500 (Thermo Fisher Scientific) size standards under denaturing conditions on an ABI3130xl Genetic Analyzer and subsequently analyzed with GeneMapper software (Thermo Fisher Scientific).

### 4.8. Variant Classification

Clinical classifications of the variants studied were performed following the ACMG guidelines through the Franklin Genoox platform (https://franklin.genoox.com/, accessed on 20 October 2022). The ACMG classification system classifies variants into five classes: Class 1 (benign), class 2 (likely benign), class 3 (VUS), class 4 (likely pathogenic), and class 5 (pathogenic) [46]. Following the outcomes of minigene assays, the PS3 criterion was used as supporting pathogenic evidence when in vitro functional studies supported a damaging effect on splicing. Conversely, the BS3 criterion was used as supporting benign evidence when minigene assays showed no splicing defects.

## Data Availability

All data relevant to the study are included in the article.

## Supplementary Materials

The following supporting information can be downloaded at: https://www.mdpi.com/article/10.3390/ijms24021562/s1.
