# Supplementary material for: Minigene Splicing Assays and Long-Read Sequencing to Unravel Pathogenic Deep-Intronic Variants in PAX6 in Congenital Aniridia
Source: Int J Mol Sci. 2023 Jan 13;24(2):1562. doi: 10.3390/ijms24021562 (PMC9863980; doi:10.3390/ijms24021562)
Supplement: Supplementary file 1 [file ijms-24-01562-s001.zip › ijms-2103814-supplementary.pdf]

Minigene splicing assays and long-read sequencing to unravel pathogenic deep-intronic variants in *PAX6* in congenital aniridia

Alejandra Tamayo [1,2,3], Gonzalo Núñez-Moreno [1,2,4], Carolina Ruiz [1], Julie Plaisancie [5,6], Alejandra Damian [1,2], Jennifer Moya [1], Nicolas Chassaing [5,6], Patrick Calvas [5,6], Carmen Ayuso [1,2], Pablo Minguez [1,2,4] and Marta Corton [1,2,*]

[1] Department of Genetics & Genomics, Instituto de Investigación Sanitaria-Fundación Jiménez Díaz University Hospital, Universidad Autónoma de Madrid (IIS-FJD, UAM), 28040 Madrid, Spain

[2] Center for Biomedical Network Research on Rare Diseases (CIBERER), Instituto de Salud Carlos III, 28029, Madrid, Spain.

[3] Department of Surgery, Medical and Social Sciences, Faculty of Medicine and Health Sciences, Science and Technology campus, University of Alcalá, 28871 Alcalá de Henares, Spain

[4] Bioinformatics Unit, Instituto de Investigación Sanitaria-Fundación Jiménez Díaz University Hospital, Universidad Autónoma de Madrid (IIS-FJD, UAM), 28240 Madrid, Spain

[5] Centre de Référence des Affections Rares en Génétique Ophtalmologique (CARGO), Hôpital Purpan, CHU Toulouse, 31000 Toulouse, France

[6] INSERM U1214, Université Toulouse III, 31000 Toulouse, France

* Correspondence: mcorton@fjd.es (M.C.)

Supplementary material

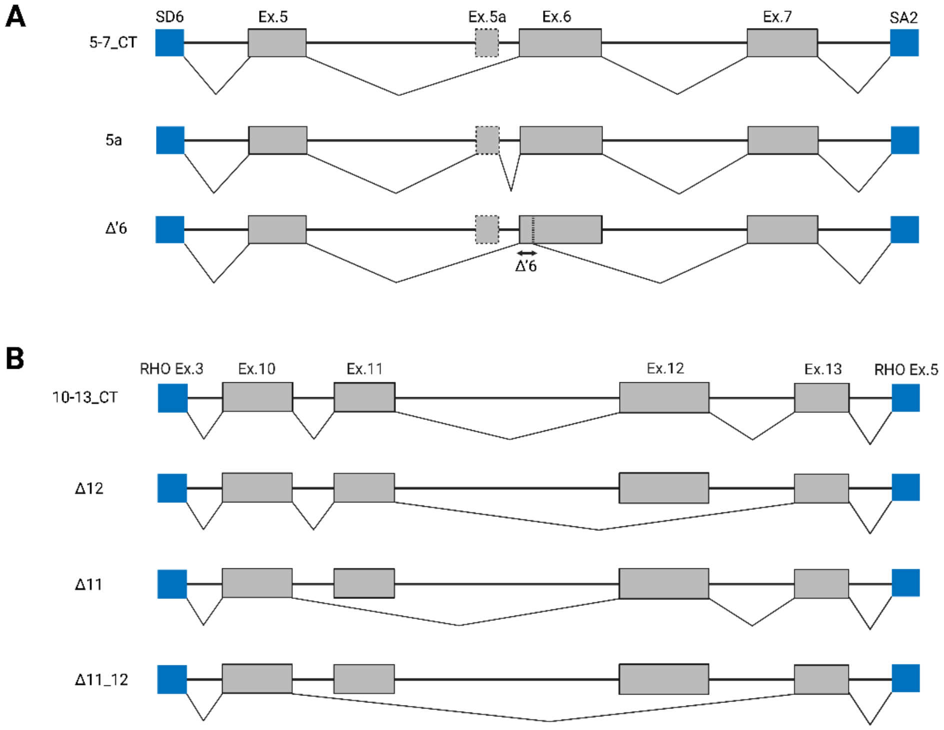

Figure S1. Splicing events observed for the wild-type minigene splicing assays. (**A**) Minigene construction for exons 5-7 yielded three major isoforms corresponding to the canonical transcript (5-7_CT), including exon 5, 6, and 7, and the two alternative isoforms, which are outlined with dashed lines: the alternative 5 exon (CT-5a) and a partially skipped exon 6 (Δ'6). (**B**) Minigene construction for exons 10-13 originated a

major isoform corresponding to the canonical transcript (10-13_CT), including exon 10, 11, 12, and 13, and three minor isoforms with skippings of exon 11 (Δ11), exon 12 (Δ12) and both exons 11-12 (Δ11_12).

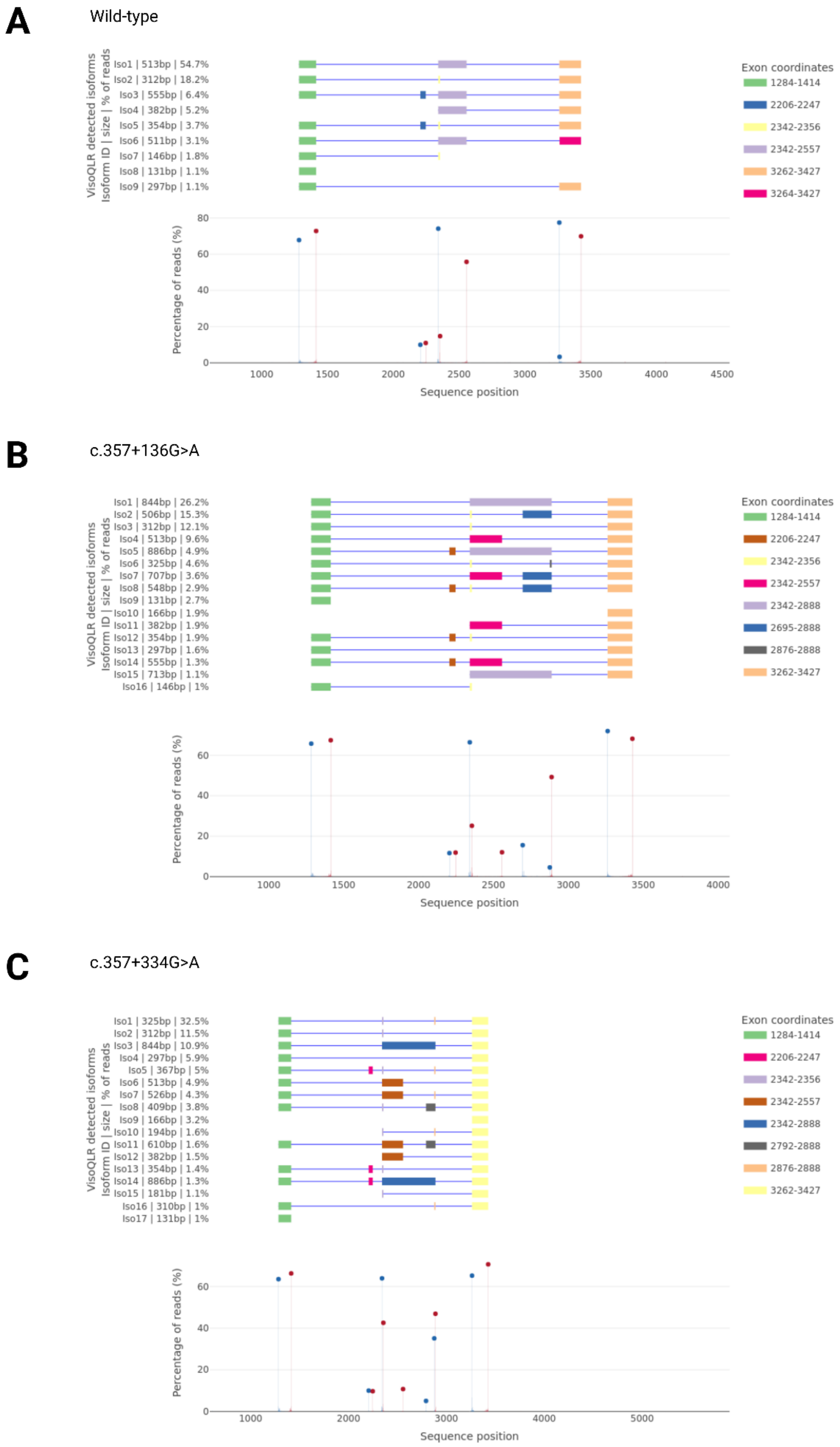

Figure S2. Long-read sequencing analysis of minigene splicing assays for *PAX6* exon 5-7 using VIsoQLR tool. The outcome for **(A)** wild-type, **(B)** c.357+136G>A, and **(C)** c.357+334G>A. Only isoforms above a threshold of 5% are shown.

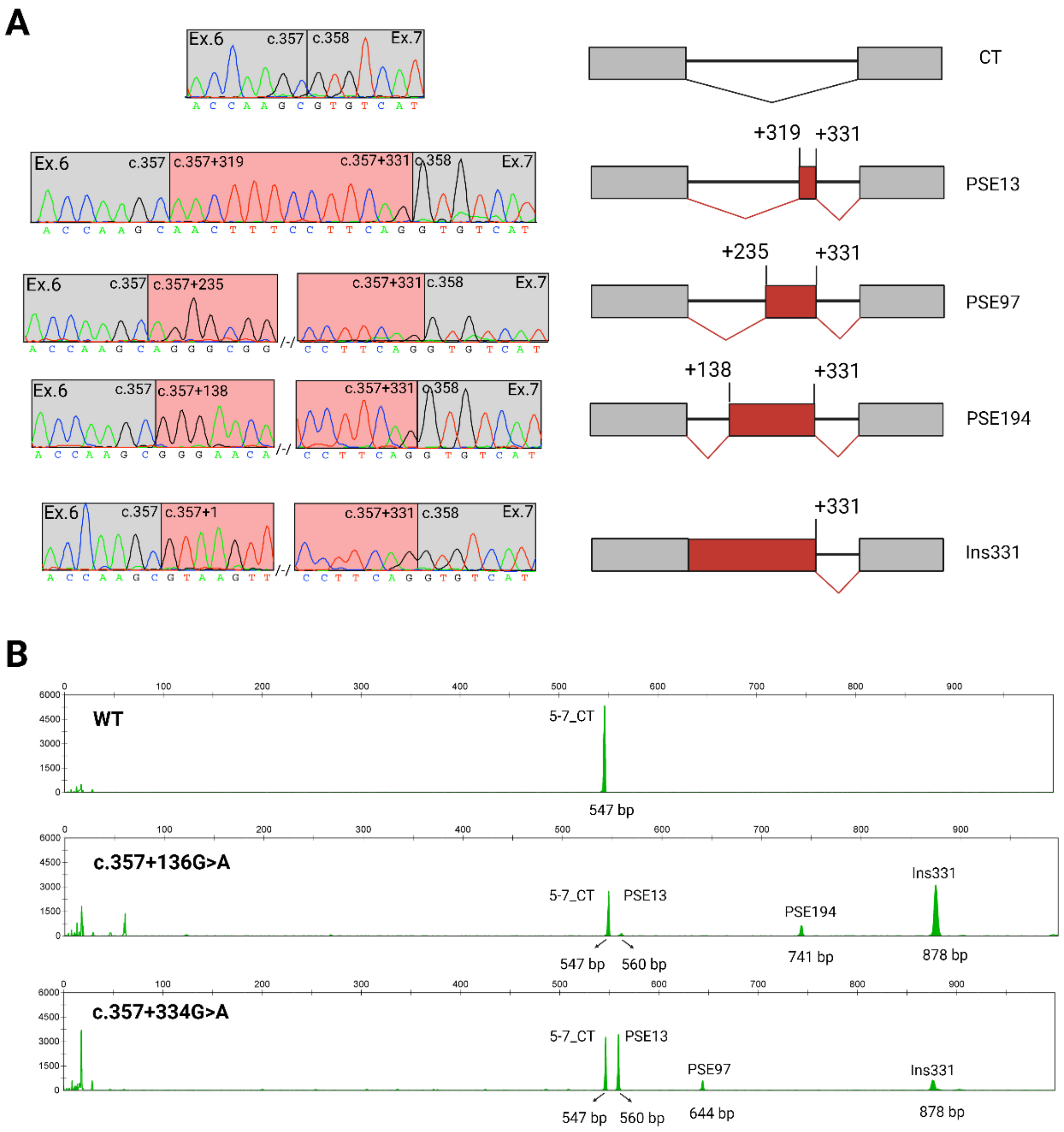

Figure S3. Validation of long-read sequencing data from minigene splicing assays for *PAX6* exons 5 to 7. (**A**) Schematic representation of the main splicing events in intron 6 arising from c.357+136G>A and c.357+334G>A (right panel) and its validation by Sanger sequencing (left panel). Wild-type exons and aberrant retentions sequences are colored gray and red, respectively. Canonical and aberrant splicing junctions are referred to as continued black or red lines, respectively. (**B**) Semi-quantitative electropherograms validation for PAX6_5-7 WT and mutant minigenes. Fluorescent-labeled primers were used, and amplicons were analyzed by capillary electrophoresis. Relative fluorescent units (RFU) and isoform sizes (bp) are represented on the y- and x-axis, respectively. Fragment sizes (bp) are indicated for each peak. Δ: deletion. CT: canonical transcript. PSE: pseudoexon insertion. Ins: insertion.

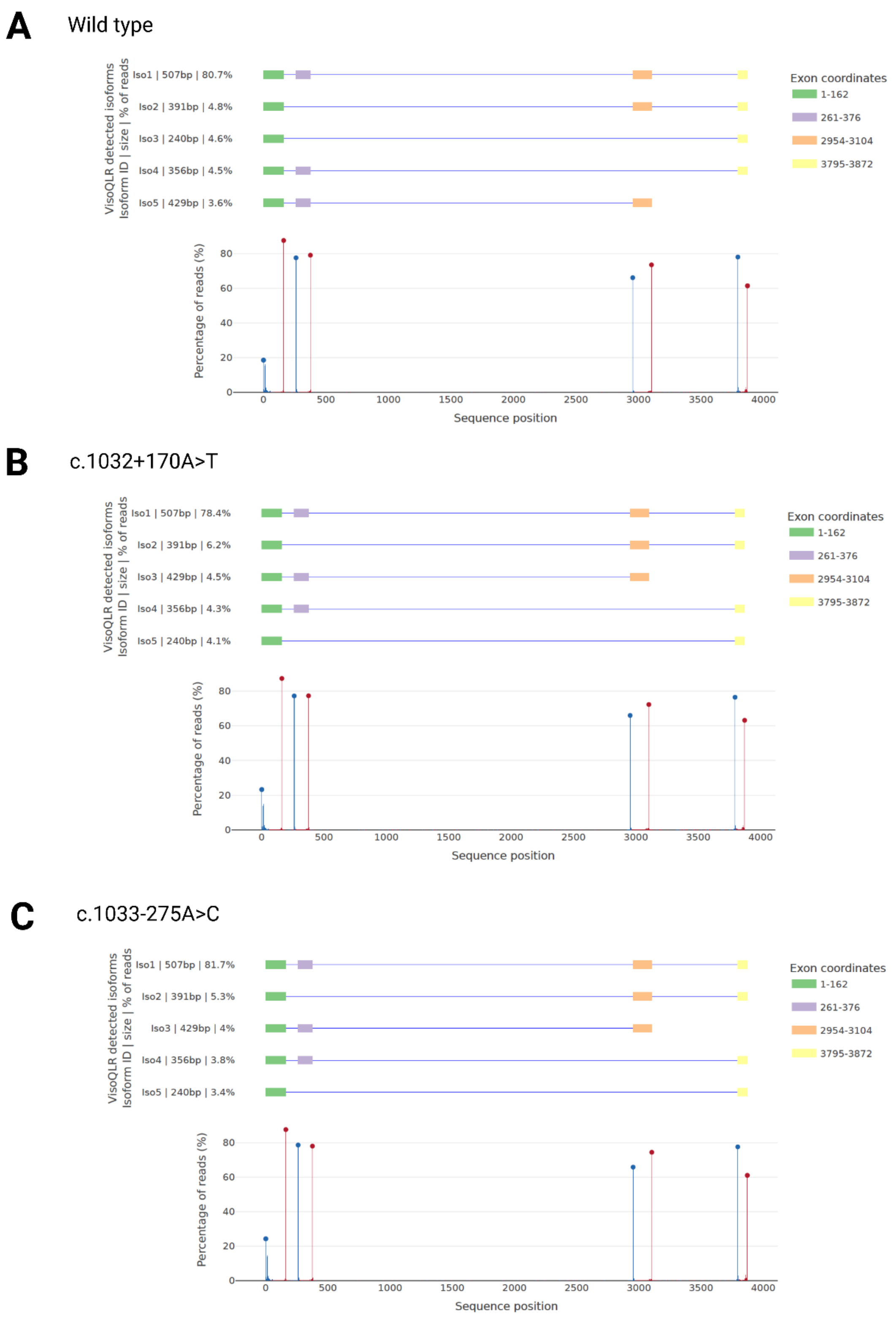

Figure S4. Long-read sequencing analysis of minigene splicing assays for *PAX6* exon 10-13 using VIsoQLR tool. The outcome for **A.** wild-type, **B.** c.1032+170A>T, and **C.** c.1033-275A>C. Only isoforms above a threshold of 5% are shown.

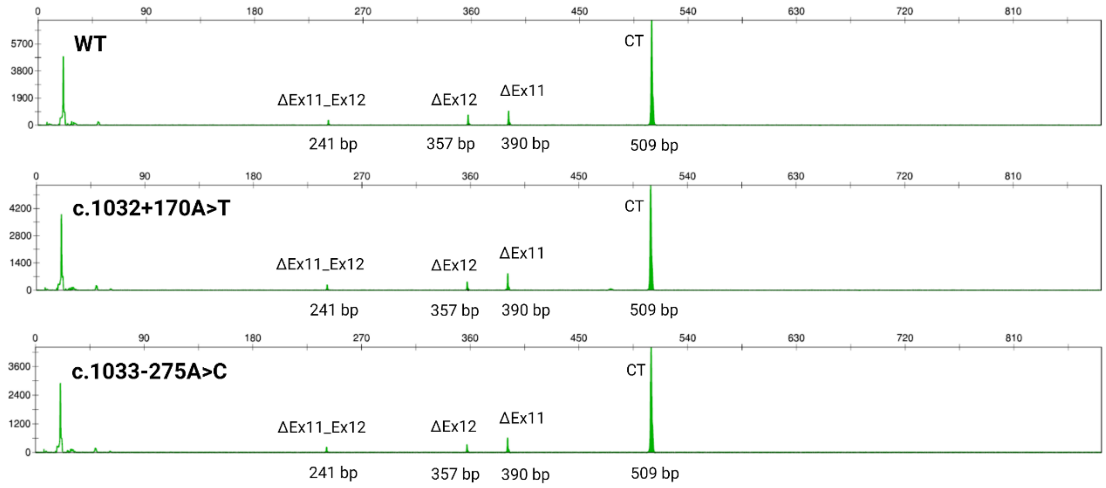

Figure S5. Validation of long-read sequencing data from minigene splicing assays for PAX6 exons 10 to 13. Semi-quantitative electropherograms of isoforms pattern belonging to wild-type, c.1032+170A>T and c.1033-275A>C. Fluorescent-labeled primers were used, and amplicons were analyzed by capillary electrophoresis. Relative fluorescent units (RFU) and isoform sizes (bp) are represented on the y- and x-axis, respectively. Fragment sizes (bp) are indicated for each peak. Δ: deletion. CT: canonical transcript. Ex: exon.

Primer name	Sequence
**Genomic amplification**	
PAX6_5F_mini	*TAT*CTCGAG**GGATCC**CTGGTGGTCCTGTTGTCCTT
PAX6_7R_mini	*AT*GATATC**ATTAAT**ACCTTCATACCGCTCCTGAC
GT_PAX6_Ex10_F	GGGGACAAGTTTGTACAAAAAAGCAGGCTGTAGACACAGTGCTAACCTG
GT_PAX6_Ex13_R	GGGGACCACTTTGTACAAGAAAGCTGGGTAAGCTCAACTGTTGTGTCCC
**Site-Directed mutagenesis**	
PAX6_c.357+136GxA_F	TCTCTGTTCCCCTAGGTACAAAGGAGACAAATGTGG
PAX6_c.357+136GxA_R	CCACATTTGTCTCCTTTGTACCTAGGGGAACAGAGA
PAX6_c.357_334GxA_F	CCAGAACTTTCCTTCAGGTATCACACATCCATTTCCA
PAX6_c.357_334GxA_R	TGGAAATGGATGTGTGATACCTGAAGGAAAGTTCTGG
PAX6_c.1032+170AxT_F	CAATCCCATCACTTTTAAGTGTTTGACTCATTAATTCATGCCC
PAX6_c.1032+170AxT_R	GGGCATGAATTAATGAGTCAAACACTTAAAAGTGATGGGATTG
PAX6_c.1033-275AxC_F	AGGTTAGAGACAAAAATCCTATTCATTTATGGATAGTGGCAACCATC
PAX6_c.1033-275AxC_R	GATGGTTGCCACTATCCATAAATGAATAGGATTTTTGTCTCTAACCT
**RNA analysis**	
SD6_F	TCTGAGTCACCTGGACAACC
SA2_R	ATCTCAGTGGTATTTGTGAGC
c.148_F	AACGGATGTGTGAGTAAAATTCTG
Rho_Ex3_PAX6_Ex10_F	CCGTCAAGGAGGTATGGTTTTC
PAX6_RNA_Ex13_R	ATCTTGGCCAGTATTGAGAC

Table S1. Primers used for genomic amplification, site-directed mutagenesis, and RNA analysis. Restriction enzyme cutting sites (XhoI-underlined and BamHI-bolded in PAX6_5F_mini and EcoRV-underlined and VspI-bolded in PAX6_7R_mini) are colored in green. Italic sequences are extra bases to facilitate enzyme cuts. Gateway recombination sequences are colored in blue.
